# Cullin3 promotes breast cancer cells metastasis and epithelial-mesenchymal transition by targeting BRMS1 for degradation

**DOI:** 10.18632/oncotarget.5999

**Published:** 2015-10-16

**Authors:** Xiongwei Huo, Suoni Li, Tingting Shi, Aili Suo, Zhiping Ruan, Hui Guo, Yu Yao

**Affiliations:** ^1^ Department of General Surgery, First Affiliated Hospital of Xi'an Jiaotong University, Xi'an, Shaanxi, 710061, China; ^2^ Department of Oncology, Shaanxi Province Tumor Hospital, Xi'an, Shaanxi, 710061, China; ^3^ Department of Oncology, First Affiliated Hospital of Xi'an Jiaotong University, Xi'an, Shaanxi, 710061, China

**Keywords:** cullin3, epithelial-mesenchymal transition, invasion, BRMS1, breast cancer

## Abstract

Metastasis is the leading cause of death in breast cancer (BC) patients. However, until now, the mechanisms of BC metastasis remain elusive. Cullin3 is a highly conserved Cullin family member present in the genomes of all eukaryotes, which has been proposed as an oncogene in many types of tumors; however, its role and underlying mechanisms in BC remain unclear. Here we show that Cullin3 is elevated in BC and its expression level is positively correlated with metastasis. Overexpression of Cullin3 in BC cells increased proliferation, epithelial-mesenchymal transition, migration and invasion *in vitro*, and enhanced tumorigenic and metastatic capacities *in vivo*. In contrast, silencing Cullin3 in aggressive and invasive BC cells inhibited these processes. Mechanistically, we found Cullin3 exerts its function through promoting BRMS1 protein degradation, which was associated with EMT, migration and invasion. BRMS1 overexpression blocked Cullin3-driven EMT, and metastasis. Our results, for the first time, portray a pivotal role of Cullin3 in stimulating metastatic behaviors of BC cells. Targeting Cullin3 may thus be a useful strategy to impede BC cell invasion and metastasis.

## INTRODUCTION

Breast cancer (BC) is the most frequently diagnosed cancer among females [1]. The incidence of BC has dramatically increased in Western countries and has become more prevalent in Asian countries [2]. Despite the advances that have been made in the treatment of BC, metastasis is the leading cause of death in BC patients [3]. Metastasis is a multistep process that results from genetic alterations, including the activation of oncogenes and the loss of function of tumor suppressors in breast cancer [4]. Despite a considerable amount of research, very few stable biomarkers have been identified for risk assessment or predication of clinical outcome in BC metastasis and further investigations are necessary. Therefore, it remains clinically important to identify novel prognostic biomarkers to improve the diagnosis and treatment of BC patients.

The vast majority of BC patients succumb to their disease as a result of metastasis [5]. Currently, treatment options for metastatic BC are limited and ineffective. Therefore, tremendous effort has been focused on the understanding of the mechanisms by which metastasis occurs in order to provide a more rational approach in the development of future metastatic BC treatments [6]. However, how metastases are formed remains less understood. Mounting evidence shows that in epithelial cancers, including BC, induction of epithelial-mesenchymal transition (EMT) is a major event that provides mobility to cancer cells in order to generate metastases [5]. EMT is characterized by the loss of epithelial characteristics and acquisition of a mesenchymal phenotype, which confers the ability for cancer cells to invade adjacent tissue and migrate to distant sites, where these cancer cells proliferate to generate new tumors [7]. Hence, clarifying the regulation of proliferation and EMT will greatly benefit our understanding of BC metastasis.

Recent research has firmly established Cullin3 as the molecular scaffold of a major class of CRLs controlling different developmental and stress responses as well as human pathologies [8]. Cullin3 is a highly conserved Cullin family member present in the genomes of all eukaryotes [8]. At the structural level, Cullin3 acts with BTB/POZ domain proteins, which function as substrate-specific adaptors. They bind Cullin3 via the BTB domain, and commonly direct substrate specificity through an independent additional protein-protein interaction domain [8]. Given the importance of Cullin3 in controlling different cellular and developmental processes, it is perhaps of little surprise that they are also linked to the pathology of various human diseases, including metabolic disorders, muscle and nerve degeneration, but also neoplastic diseases [9–11]. Rapid degradation of Nrf2 by Cullin3 provides a molecular basis for induction of cytoprotective enzymes both in response to stress and during disease development [12]. Genetic disruptions of the Cullin3 complex found in lung cancer patients, at both copy number and gene expression levels, lead to upregulation of IKKβ protein levels and activation of the NF-κB signaling pathway [13]. Similarly, regulation of Cullin3 protein levels has been linked to bladder cancer aggressiveness and to liver tumorigenesis [9, 14, 15]. Likewise, Cullin3 impinges on hypoxia, an essential feature of the solid tumour environment [9]. Thus, Cullin3 may act as an oncogene, but whether Cullin3 plays a role in BC formation and metastasis remains unknown.

In this study, we found that Cullin3 expression was significantly upregulated in breast cancer lesions compared with paired normal breast tissues and its expression level is positively correlated with metastasis. Additionally, we present the evidence that Cullin3 ectopic expression promotes BC cells growth, invasion, metastasis and EMT. While, by silencing Cullin3 in BC cells could inhibit all of these effects. These functional effects of Cullin3 were exerted through control of BRMS1 degradation. The down-regulation of BRMS1 triggered by Cullin3 therefore enforces BC cells oncogenesis and metastasis. For the first time, we portray a pivotal role of Cullin3 in regulating metastatic behaviors of BC cells. Our findings provide a novel mechanistic role of Cullin3 in BC metastasis, suggesting that Cullin3 may serve as a potential therapeutic target for advanced BCs.

## RESULTS

### Cullin3 is highly expressed and correlated with distant metastasis in BC

**Figure 1:**
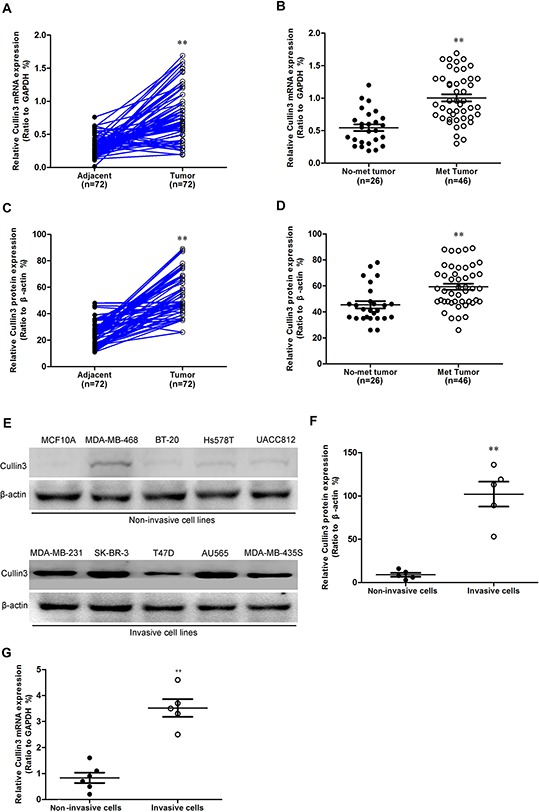
Cullin3 is highly expressed in breast cancer **A.** Cullin3 mRNA expression was analyzed by quantitative RT-PCR in breast tumors and adjacent tissues. **B.** comparison of the expression levels of Cullin3 mRNA in not metastasis and metastatic BC tissues. **C.** comparison of the relative expression levels of Cullin3 protein in breast tumors and adjacent tissues. **D.** comparison of the relative expression levels of Cullin3 protein in not metastasis and metastatic BC tissues. **E.** Cullin3 protein expression was analyzed by Western blot in breast cancer cell lines. **F.** comparison of the relative expression levels of Cullin3 protein in breast cancer cell lines. **G.** Cullin3 mRNA expression was analyzed by qRT-PCR in breast cancer cell lines. **P < 0.01 is based on the Student *t* test. All results are from three independent experiments. Error bars, SD.

To investigate whether Cullin3 might be involved in BC, the mRNA expression level of Cullin3 in BC tissues and its matched normal adjacent tissues was determined by qRT-PCR in 72 samples (Supplemental Figure 1). As compared with normal tissues, BC specimens showed overexpression of Cullin3 (Figure 1A). We then analyzed Cullin3 expression in BCs without or with distant metastasis; we found that Cullin3 mRNA overexpression was significantly correlated with distant metastasis in BC tissues (Figure 1B). The protein level of Cullin3 in these tissue samples were also analyzed by western blot (Supplemental Figure 2). The protein level of Cullin3 was upregulated in BCs samples as compared with the normal adjacent tissues samples (Figure 1C). Furthermore, we found that Cullin3 protein expression was also significantly correlated with distant metastasis in BC tissues (Figure 1D). As showed in Figure 1E, 1F, and 1G, the expression level of Cullin3 protein and mRNA in invasive BC cell lines was higher than that in the non-invasive BC cell lines. These data demonstrated that the upregulation of Cullin3 might be relevant to development and invasive of BC.

**Figure 2:**
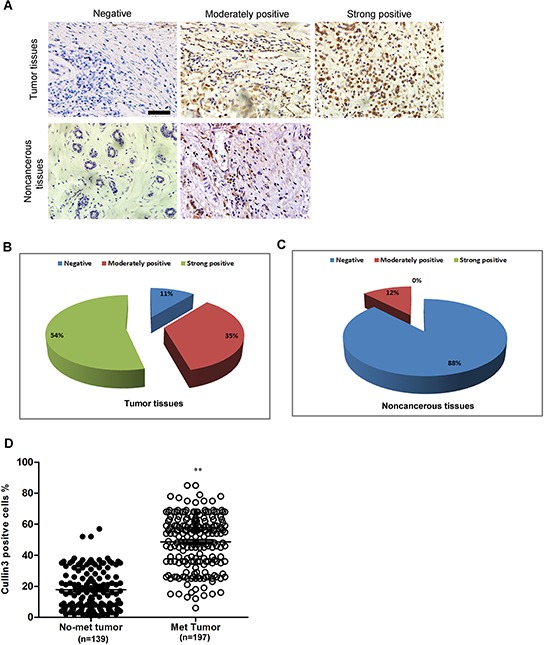
Cullin3 is correlated with distant metastasis in breast cancer **A.** Cullin3 protein expression was analyzed by immunohistochemical analysis in 336 cases BC tissues and the representative results were shown. **B.** the percentage of negative, moderately positive and strong positive expression of Cullin3 in breast cancer tissues was analyzed. **C.** the percentage of negative, moderately positive and strong positive expression of Cullin3 in normal breast tissues was analyzed. **D.** the association between Cullin3 expression in breast cancer and the survival time of selected patients was analyzed with Kaplan-Meier survival analysis. **P < 0.01 is based on the Student *t* test. All results are from three independent experiments. Error bars, SD. Scale bars, 50 μm (upper) in A

We examined Cullin3 protein expression in more BC samples by IHC (Figure 2A). We observed that the level of Cullin3 positive cells was markedly higher in BC tissues than the level in the normal breast tissues (Figure 2B and 2C). Most importantly, Cullin3 overexpression was consistently significantly correlated to distant metastasis in these BC samples (Figure 2D). To investigate the relationship between Cullin3 expression and clinicopathological parameters in the 336 cases with BCs, these cases were first divided into two subgroups: “Cullin3 negative” and “Cullin3 positive” as defined in the immunohistochemistry section of “Materials and methods”. Significant correlations were found between Cullin3 expression and tumor diameter and lymph node metastasis. There were no statistical connections between Cullin3 expression and the rest clinicopathological parameters, such as patient age, estrogen receptor, and progesterone receptor (Supplemental Table 1). These results collectively indicate a functional role of Cullin3 in aggressive behaviors of BCs.

### Cullin3 promotes migratory and invasive capacities of BC cells *in vitro*

**Figure 3:**
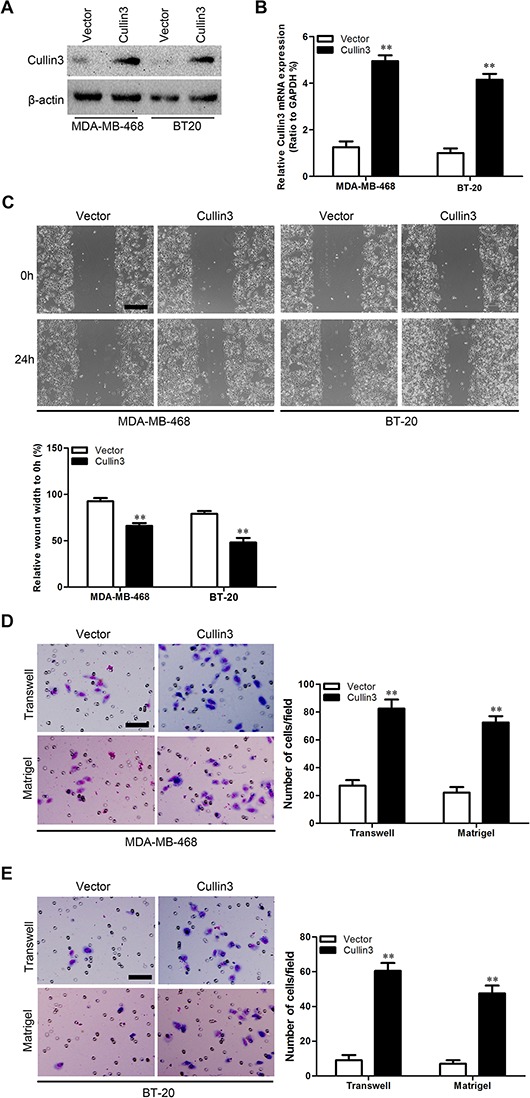
Ectopic expression Cullin3 promotes migratory and invasive capacities of BC cells *in vitro* **A.** expression level of Cullin3 protein was measured by Western blotting in established cell lines. **B.** expression level of Cullin3 mRNA was measured by qRT-PCR in established cell lines. **C.** MDA-MB-468-Cullin3, BT-20-Cullin3 and theirs control vector cells were subjected to wound healing assays; the uncovered areas in the wound healing assays were quantified as a percentage of the original wound area. **D.** MDA-MB-468-Cullin3 and its control vector cells were subjected to Transwell migration (top), and Matrigel invasion assays (bottom), quantification of migrated cells through the membrane and invaded cells through Matrigel of each cell line are shown as proportions of their vector controls. **E.** BT-20-Cullin3 and its control vector cells were subjected to Transwell migration (top), and Matrigel invasion assays (bottom), quantification of migrated cells through the membrane and invaded cells through Matrigel of each cell line are shown as proportions of their vector controls. **P < 0.01 is based on the Student *t* test. All results are from three independent experiments. Error bars, SD.

**Figure 4:**
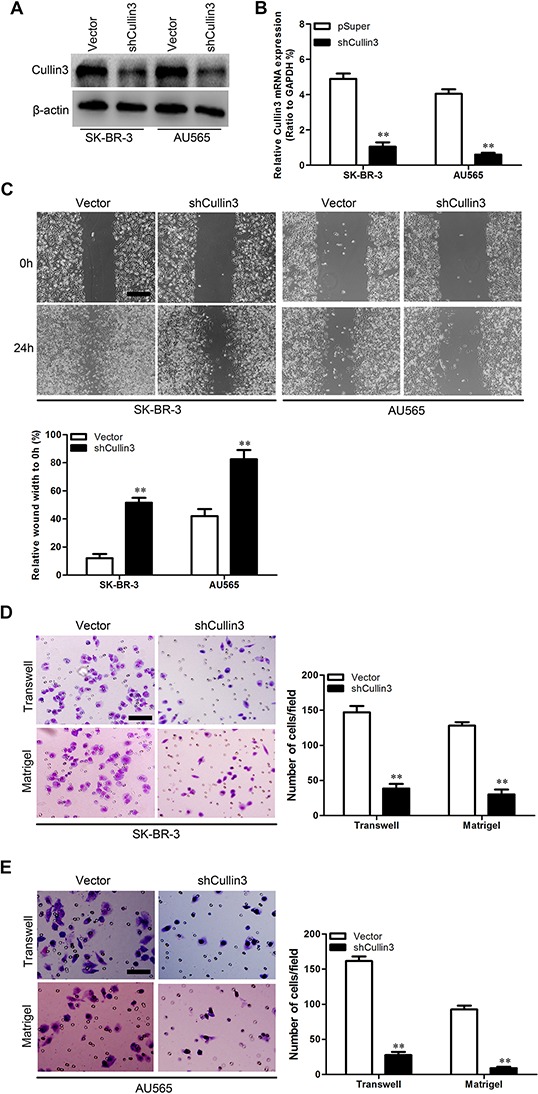
Knocking down Cullin3 inhibits migratory and invasive capacities of BC cells *in vitro* **A.** expression level of Cullin3 protein was measured by Western blotting in established cell lines. **B.** expression level of Cullin3 mRNA was measured by qRT-PCR in established cell lines. **C.** SK-BR-3-shCullin3, AU565-shCullin3 and theirs control vector cells were subjected to wound healing assays; the uncovered areas in the wound healing assays were quantified as a percentage of the original wound area. **D.** SK-BR-3-shCullin3 and its control vector cells were subjected to Transwell migration (top), and Matrigel invasion assays (bottom), quantification of migrated cells through the membrane and invaded cells through Matrigel of each cell line are shown as proportions of their vector controls. **E.** AU565-shCullin3 and its control vector cells were subjected to Transwell migration (top), and Matrigel invasion assays (bottom), quantification of migrated cells through the membrane and invaded cells through Matrigel of each cell line are shown as proportions of their vector controls. **P < 0.01 is based on the Student *t* test. All results are from three independent experiments. Error bars, SD.

In order to test the oncogenic activity of Cullin3 in BC, we retrovirally established stable overexpression of Cullin3 in MDA-MB-468 and BT-20 cells, and silencing of Cullin3 in SK-BR-3 and AU565 cells. The levels of Cullin3 in these resultant cell lines were verified by western blotting (Figure 3A and Figure 4A) and qRT-PCR (Figure 3B and Figure 4B). The effect of Cullin3 on cell migration was first assessed by wound healing assay. Both MDA-MB-468-Cullin3 and BT-20-Cullin3 cells had significantly faster closure of the wound area compared to their control cells (Figure 3C). This result was confirmed by Boyden's chamber assay (Figure 3D and 3E). Moreover, MDA-MB-468-Cullin3 and BT-20-Cullin3 cells showed a greater degree of invasion through Matrigel (Figure 3D and 3E). In contrast, silencing Cullin3 dramatically reduced the migratory and invasive capacity of SK-BR-3 and AU565 cells (Figure 4C–4E). These results indicate that Cullin3 promotes migratory and invasive behaviors in BC cells.

### Cullin3 promotes metastasis *in vivo*

**Figure 5:**
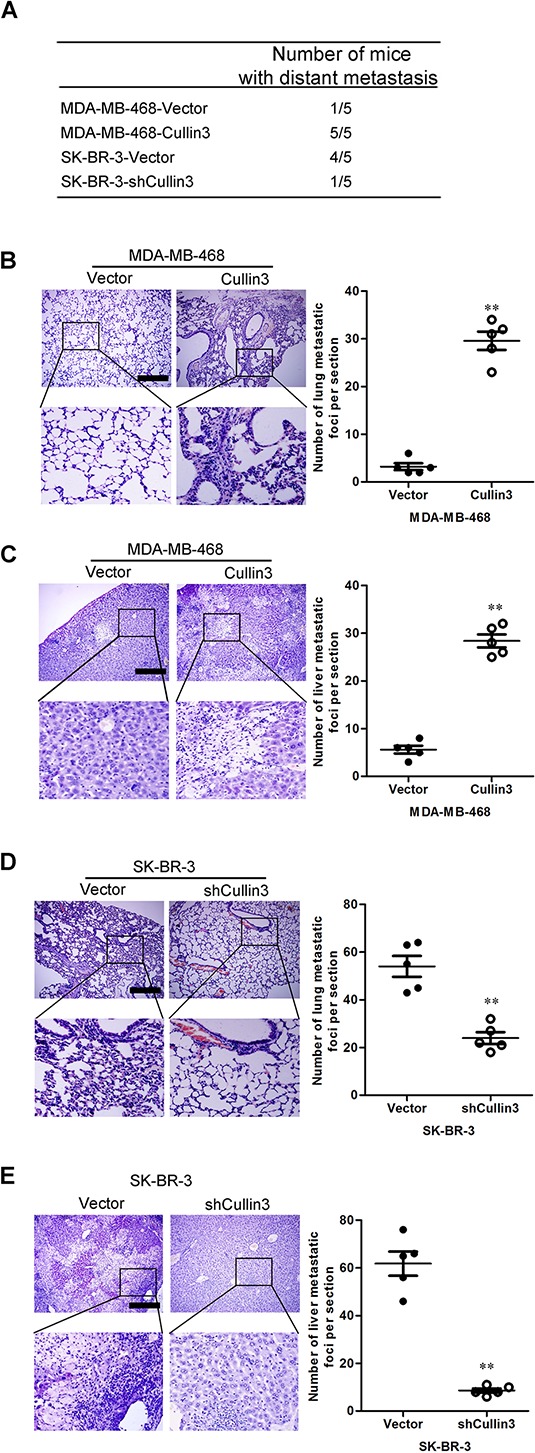
CUL4A promotes metastasis of human breast cancer cells **A.** the total numbers of mice with distant metastasis at 60 days after injection of MDA-MB-468-Cullin3, SK-BR-3-shCullin3, or their respective control cells into tail vein were analyzed. **B.** the numbers of metastatic foci per section in lung of mouse with injection of MDA-MB-468-Cullin3 or its control cells were analyzed. **C.** the numbers of metastatic foci per section in liver of mouse with injection of MDA-MB-468-Cullin3 or its control cells were analyzed. **D.** the numbers of metastatic foci per section in lung of mouse with injection of SK-BR-3-shCullin3 or its control cells were analyzed. **E.** the numbers of metastatic foci per section in liver of mouse with injection of SK-BR-3-shCullin3 or its control cells were analyzed. **P < 0.01 is based on the Student *t* test. All results are from three independent experiments. Error bars, SD.

We then investigated the functional relevance of Cullin3 for metastasis *in vivo*. MDA-MB-468-Cullin3, SK-BR-3-shCullin3 and their corresponding control cells were injected into nude mice through the tail vein. Cullin3 overexpression not only significantly increased the number of mice with distant metastasis (Figure 5A), but also dramatically increased the number of metastatic tumors in both lung and liver of each mouse (Figure 5B and 5C). Silencing Cullin3 in SK-BR-3 cells inhibited metastatic behavior, both in terms of the number of mice with distant metastasis (Figure 5A) and the number of metastatic tumors in the lung and liver of each mouse (Figure 5D and 5E). Therefore, the *in vivo* results further demonstrate the critical role of Cullin3 in BC metastasis.

### Cullin3 promotes proliferative capacity of BC cells

Compared to vector-only controls, both MDA-MB-468-Cullin3 and BT-20-Cullin3 cells had significant increases in cell proliferation by MTT assay (Supplemental Figure 3A and 3B). In contrast, silencing of Cullin3 in SK-BR-3 and AU565 cells significantly reduced cell proliferation (Supplemental Figure 3C and 3D). To extend our *in vitro* observations, we investigated whether Cullin3 could regulate tumorigenic and metastatic capacity of BC cells *in vivo*. MDA-MB-468-Cullin3, SK-BR-3-shCullin3 and their corresponding control cells were subcutaneously injected into nude mice. Tumor size was measured every week up to 6 weeks. As expected, the tumors from MDA-MB-468-Cullin3 cells grew more rapidly at the implantation site than the control cells (Supplemental Figure 3E–3G). In contrast, silencing Cullin3 in the typically aggressive SK-BR-3 cells led to a dramatic decrease in tumor volume and weight (Supplemental Figure 3H–3J). Taking together, these results suggest that Cullin3 is an important regulator of proliferation in BC cells.

### Cullin3 regulates the transition between epithelial and mesenchymal phenotypes in BC cells

**Figure 6:**
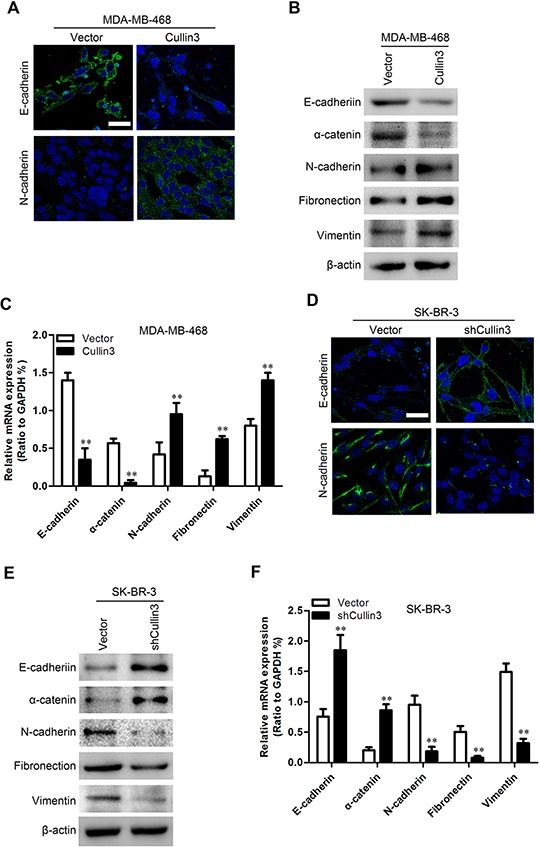
Cullin3 regulates the transition between epithelial and mesenchymal phenotypes in BC cells **A.** expression of E-cadherin and N-cadherin was analyzed by immunofluorescence stains in MDA-MB-468-Cullin3 and its control cells. **B.** expression of epithelial and mesenchymal marker was analyzed by Western blotting in MDA-MB-468-Cullin3 and its control cells. **C.** expression of epithelial and mesenchymal marker was analyzed by qRT-PCR in MDA-MB-468-Cullin3 and its control cells. **D.** expression of E-cadherin and N-cadherin was analyzed by immunofluorescence stains in SK-BR-3-shCullin3 and its control cells. **E.** expression of epithelial and mesenchymal marker was analyzed by Western blotting in SK-BR-3-shCullin3 and its control cells. **F.** expression of epithelial and mesenchymal marker was analyzed by qRT-PCR in SK-BR-3-shCullin3 and its control cells. **P < 0.01 is based on the Student *t* test. All results are from three independent experiments. Error bars, SD.

To investigate whether Cullin3 positively regulates cell migration and invasion, we first observed the morphological changes and found that both MDA-MB-468-Cullin3 and BT-20-Cullin3 cells exhibited fibroblastic morphology (Supplemental Figure 4A and 4B). This observation was further confirmed by expression analyses of epithelial and mesenchymal markers. We showed that Cullin3 overexpression decreased the levels of epithelial markers (E-cadherin and α-catenin) and increased the levels of mesenchymal markers (N-cadherin, Fibronectin and vimentin) in both cell lines (Figure 6A–6C, Supplemental Figure 5A–5C). Conversely, both SK-BR-3-shCullin3 and AU565-shCullin3 cells reverted to an epithelial phenotype as compared to their respective control cells (Supplemental Figure 4C and 4D). Consistent with this, silencing Cullin3 increased levels of epithelial markers, and decreased levels of mesenchymal markers (Figure 6D–6F, Supplemental Figure 5D–5F). Taken together, these findings suggest that Cullin3 plays an important role in regulating EMT-MET plasticity of BC cells.

### Cullin3 regulates BRMS1 expression through degradation

**Figure 7:**
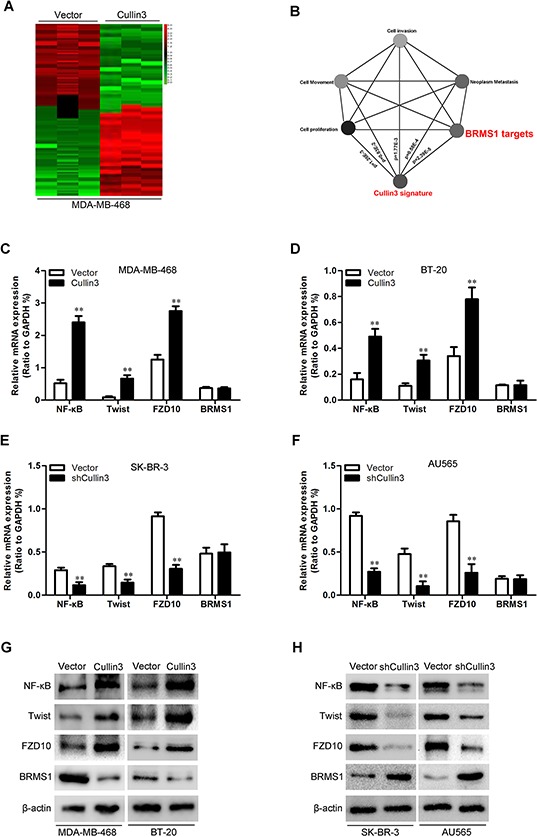
Cullin3 downregulates BRMS1 expression in BC cells **A.** supervised hierarchical clustering of the genes differentially expressed after Cullin3 overexpression in MDA-MB-468 cells. **B.** gene set enrichment analysis was carried out using ConceptGen. **C.** the mRNA levels of NF-κB, Twist, FZD10 and BRMS1 were analyzed by qRT-PCR in MDA-MB-468-Cullin3 and its control cells. **D.** the mRNA levels of NF-κB, Twist, FZD10 and BRMS1 were analyzed by qRT-PCR in BT-20-Cullin3 and its control cells. **E.** the mRNA levels of NF-κB, Twist, FZD10 and BRMS1 were analyzed by qRT-PCR in SK-BR-3-shCullin3 and its control cells. **F.** the mRNA levels of NF-κB, Twist, FZD10 and BRMS1 were analyzed by qRT-PCR in AU565-shCullin3 and its control cells. **G.** the protein levels of NF-κB, Twist, FZD10 and BRMS1 were analyzed by Western blotting in MDA-MB-468-Cullin3, BT-20-Cullin3 and theirs control cells. **H.** the protein levels of NF-κB, Twist, FZD10 and BRMS1 were analyzed by Western blotting in SK-BR-3-shCullin3, AU565-shCullin3 and theirs control cells. **P < 0.01 is based on the Student *t* test. All results are from three independent experiments. Error bars, SD.

To better understand the mechanisms by which Cullin3 engaged in BC development and progression, we performed gene expression profiling on MDA-MB-468-Cullin3 and its control cells. Microarray analyses identified a list of genes significantly differentially expressed after Cullin3 overexpression including downregulation of BRMS1 target genes (Figure 7A). Furthermore, gene set enrichment analysis indicated that proliferation, neoplasm metastasis and invasion, cell movement and motility, and BRMS1 related gene signatures were significantly changed in Cullin3 overexpression cells (Figure 7B), and supporting the idea that Cullin3 regulates proliferation, EMT and cancer invasion and metastasis. These data also led us to hypothesize that Cullin3 exerts these functions possibly via BRMS1. To test this, we first determined whether BRMS1 is a downstream target of Cullin3 in BC cells. Expression of BRMS1 and its target genes (NF-κB, Twist and FZD10) [19, 20] in the cells with altered Cullin3 expression were further evaluated by qRT-PCR and Western blotting. MDA-MB-468-Cullin3 and BT-20-Cullin3 cells exhibited greatly increased BRMS1 target genes (NF-κB, Twist and FZD10) both mRNA and protein levels (Figure 7C, 7D and 7G), and silencing Cullin3 in SK-BR-3 and AU565 cells dramatically decreased these mRNA and protein levels (Figure 7E, 7F and 7H). While the expression of BRMS1 just modulated by Cullin3 change in protein level. Suggesting the regulation of BRMS1 expression by Cullin3 is at post-transcriptional level.

**Figure 8:**
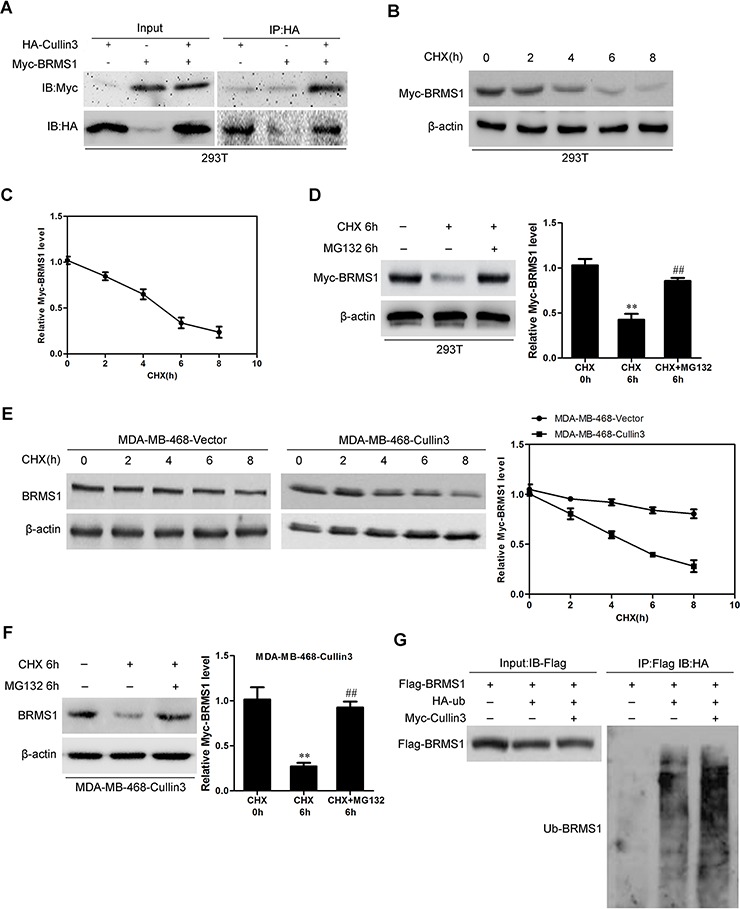
Cullin3 regulates BRMS1 expression through degradation **A.** 293T cells were co-transfected with HA-tagged Cullin3 and Myc-tagged BRMS1 constructs. Immunoprecipitation and subsequent immunoblot analysis revealed that HA-tagged Cullin3 co-immunoprecipitated with Myc-tagged BRMS1. **B.** and **C.** the half-life period of ectopic BRMS1 in 293T cells were analyzed by Western blotting. **D.** treatment with the proteasome inhibitor MG132 markedly reversed the BRMS1 degradation. **E.** the half-life period of BRMS1 in MDA-MB-468-Cullin3 and its control cells were measured by Western blot. **F.** treatment with the proteasome inhibitor MG132 markedly decreased the BRMS1 degradation in MDA-MB-468-Cullin3 cells. **G.** ectopic expression of Myc-Cullin3 and Flag-BRMS1 significantly increased polyubiquitylated BRMS1 in 293T cells. ^**^, ^##^, *P* < 0.01 is based on the Student *t* test. All results are from three independent experiments. Error bars, SD.

To understand how Cullin3 regulates BRMS1 expression at the post-transcriptional level. 293T cells were co-transfected with HA-tagged Cullin3 and Myc-tagged BRMS1 constructs. Immunoprecipitation and subsequent immunoblot analysis revealed that HA-tagged Cullin3 co-immunoprecipitated with Myc-tagged BRMS1, confirming a physical interaction between the two proteins (Figure 8A). The physical interaction between BRMS1 and Cullin3 suggested that Cullin3 could target BRMS1 for ubiquitination and subsequent degradation. Subsequently, we measured the half-life period of ectopic BRMS1 in 293T cells. As shown in Figure 8B and 8C, the half-life period of ectopic BRMS1 is about 5 hours. Treatment with the proteasome inhibitor MG132 markedly reversed the BRMS1 degradation (Figure 8D). To investigate whether Cullin3 is involved in the BRMS1 degradation in BC cells, the half-life period of BRMS1 in MDA-MB-468-Cullin3 and its control cells were measured by Western blot, and we found that when ectopic Cullin3 in MDA-MB-468 cells the half-life period of BRMS1 was significantly decreased (Figure 8E). We also found that treatment with the proteasome inhibitor MG132 markedly decreased the BRMS1 degradation in MDA-MB-468-Cullin3 cells. We next tested whether Cullin3 polyubiquitylates BRMS1 *in vivo*, ectopic expression of Myc-Cullin3 and Flag-BRMS1 significantly increased polyubiquitylated BRMS1 in 293T cells. Therefore, all of these results indicated that Cullin3 interacts with and directly polyubiquitylates BRMS1.

### BRMS1 is a mediator for Cullin3-induced EMT, migration, and invasion in BC cells

**Figure 9:**
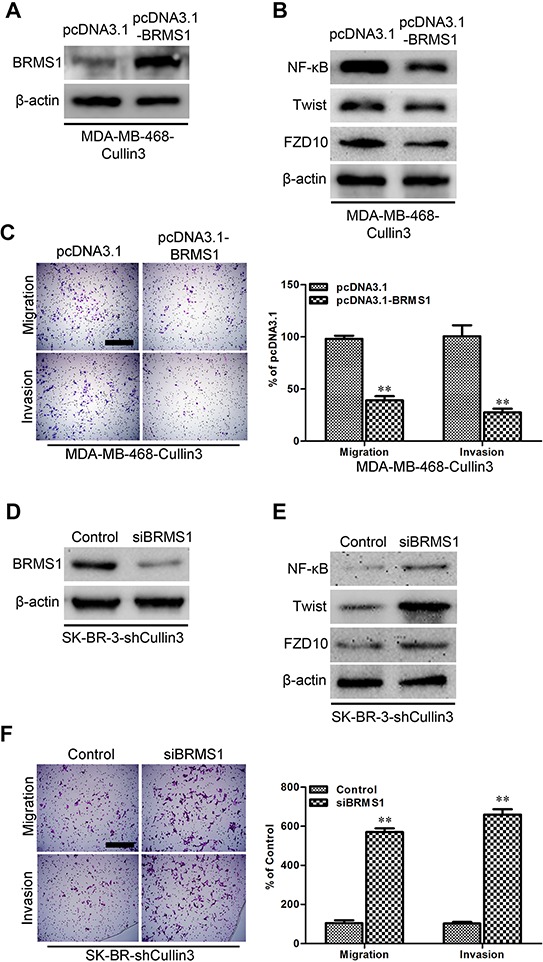
BRMS1 is a mediator for Cullin3-induced EMT, migration, and invasion in BC cells **A.** the expression of BRMS1 was analyzed by Western blotting in ectopic expression BRMS1 in MDA-MB-468-Cullin3 cells. **B.** the expression of NF-κB, Twist, and FZD10 were analyzed by Western blotting in ectopic expression BRMS1 in MDA-MB-468-Cullin3 cells. **C.** indicated cells were subjected to Transwell migration (top), and Matrigel invasion assays (bottom), quantification of migrated cells through the membrane and invaded cells through Matrigel of each cell line are shown as proportions of their vector controls. **D.** the expression of BRMS1 was analyzed by Western blotting in BRMS1 knocking down cells. **E.** the expression of NF-κB, Twist, and FZD10 were analyzed by Western blotting in BRMS1 knocking down cells. **F.** indicated cells were subjected to Transwell migration (top), and Matrigel invasion assays (bottom), quantification of migrated cells through the membrane and invaded cells through Matrigel of each cell line are shown as proportions of their vector controls. ^**^P < 0.01 is based on the Student *t* test. All results are from three independent experiments. Error bars, SD.

On the basis of the indispensable role of BRMS1 in the biologic functions of Cullin3, we overexpressed BRMS1 in MDA-MB-468-Cullin3 cells (Figure 9A) and silenced BRMS1 in SK-BR-3-shCullin3 cells (Figure 9D). Of note, BRMS1 overexpression significantly decreased NF-κB, Twist and FZD10 in MDA-MB-468-Cullin3 cells (Figure 9B). Moreover BRMS1 overexpression significantly decreased the migration, and invasion of MDA-MB-468-Cullin3 cells (Figure 9C), and reversed Cullin3-induced EMT markers changes (Supplemental Figure 6A). Meanwhile silencing BRMS1 increased NF-κB, Twist and FZD10 in SK-BR-3-shCullin3 cells (Figure 9E). Moreover silencing BRMS1 in SK-Hep1-pSuper-shCullin3 cells significantly increased the migration and invasion (Figure 9F), and reversed shCullin3-induced EMT markers changes (Supplemental Figure 6B). Taken together, these results show that BRMS1 mediates Cullin3-induced EMT, migration and invasion in breast cancer cells.

## DISCUSSION

The development of breast cancer is a complex multistep process associated with numerous genetic alterations, downregulation of tumor suppressor genes, upregulation of oncogenes and early hematogenous dissemination of tumor cells [21]. Accordingly, the elucidation of the molecular mechanisms in breast cancer has been the subject of extensive research over the past decade [22]. The high mortality rate of breast cancer is caused by frequent tumor metastasis, postsurgical recurrence, and late detection at advanced stages [23, 24]. However, good diagnostic markers, drug targets and therapeutic strategies are still insufficient for successful treatment of breast cancer.

The objective of this study was to clarify the role of Cullin3 in breast cancer. In this research we show that Cullin3 plays an important part in BC. We found that as compared with normal tissues, BC specimens showed overexpression of Cullin3 and its overexpression was significantly correlated with distant metastasis. Overexpression of Cullin3 was associated with decreased overall survival of BC patients. Cullin3 overexpression in BC cells induced proliferation, EMT, migration, invasion *in vitro* and enhanced tumorigenic and metastatic capacities *in vivo*. In contrast, silencing Cullin3 reversed these events in invasive BC cells. We also showed that a mechanistic link between Cullin3 and BRMS1 through Cullin3-mediated degradation. Overexpression of BRMS1 attenuated Cullin3 function and had effects similar to those elicited by direct silencing of Cullin3. These results lead us to propose a model for Cullin3 regulation of EMT and metastasis through regulation of BRMS1 in BCs.

Recent reports have firmly established Cullin3 as major regulator of different cellular and developmental processes as well as stress responses in both metazoans and higher plants [9]. In humans, functional alterations of Cullin3 have been associated with various pathologies, including metabolic disorders, muscle, and nerve degeneration, as well as cancer [9]. It is intriguing that the IκB-IKKβ pathway frequently misregulated in cancer is also a target of the Cullin3, and those genetic disruptions of the Cullin3 complex found in lung cancer patients, at both copy number and gene expression levels, lead to upregulation of IKKβ protein levels and activation of the NF-κB signaling pathway. Similarly, regulation of Cullin3 protein levels has been linked to bladder cancer aggressiveness and to liver tumorigenesis [8]. Likewise, Cullin3 impinges on hypoxia, an essential feature of the solid tumour environment. Under hypoxic conditions, HIF-1 pathways upregulate the BTB protein KLHL20, and the Cullin3-KLHL20 E3-ligase targets the PML protein for degradation to potentiate HIF-1 signalling and disease progression in prostate cancer [9]. In this case, Cullin3-dependent ubiquitylation requires prior sequential PML modification by CDK1/2 phosphorylation and Pin1-mediated prolyl-isomerization [7]. Consistent with this research, in the present study we showed that overexpression of Cullin3 promoted BC cells proliferation and enhanced tumor formation *in vivo*. Interestingly, our study points to a novel function of Cullin3 in BC metastasis through regulating EMT via down regulation of BRMS1.

BC cells with ectopic expression of Cullin3 displayed an EMT phenotype, including the associated stimulatory effects on migration and invasion *in vitro*. Interestingly, our results indicate that Cullin3 not only promotes EMT, but silencing of Cullin3 also leads to MET. All of these characteristics induced by Cullin3 *in vitro* culminated to increased numbers of distant metastases *in vivo*. These empirical findings provide a mechanistic framework to explain the clinical observations that BC patients with high levels of Cullin3 in tissue samples have more chance of distant metastasis, a significantly shorter overall and disease-free survival.

The roles of several transcription factors as migration regulators have been extensively reported. In our effort to elucidate the mechanism how Cullin3 modulates migration in breast cancer cells, we identified BRMS1 as an effective mediator of Cullin3-induced migration. BRMS1 is one of the most frequently mutated tumor suppressors in human cancer including breast cancer [25–27]. Mechanistically, BRMS1 reduces cell motility through a variety of pathways, such as NF-κB, Twist and FZD10 [27, 20]. The mechanistic connection between Cullin3 and BRMS1 was previously unknown. In this study, we showed that Cullin3 interacts with and directly polyubiquitylates BRMS1. We also confirmed that there is a physical interaction between the Cullin3 and BRMS1. The physical interaction between BRMS1 and Cullin3 suggested that Cullin3 could target BRMS1 for ubiquitination and subsequent degradation.

In sum, Cullin3 overexpression has been linked with poor clinical outcome and implicated in breast cancer progression. Specifically, we provide the first evidence that Overexpression of Cullin3 in BC cells increased proliferation, epithelial-mesenchymal transition, migration and invasion *in vitro*, and enhanced tumorigenic and metastatic capacities *in vivo*. In contrast, silencing Cullin3 in aggressive and invasive BC cells inhibited these processes. Mechanistically, we found Cullin3 exerts its function through modulation BRMS1 protein degradation, which was associated with EMT, migration and invasion. These studies prompt further investigation of Cullin3 as potential targets for the development of better treatment strategies for advanced invasive breast cancer.

## MATERIALS AND METHODS

### Chemicals and antibodies

Lipofectamine 2000 transfection and TRIZOL LS Reagents were purchased from Invitrogen (Grand Island, NY, USA). Antibodies against Cullin3, NF-κB, Twist and FZD10 were purchased from Abcam (Cambridge, MA, USA). E-cadherin, N-cadherin, vimentin, BRMS1, Fibronectin and β-actin antibodies were from Cell Signaling technology (Danvers, MA, USA). Anti-α-catenin antibody was from BD (Franklin Lakes, NJ, USA). Unless otherwise noted, all other chemicals were from Sigma (St. Louis, MO, USA).

### Patients and specimens

Seventy-two breast tumor and para-cancerous tissues which, were used for qRT-PCR and Western blot analysis, were randomly collected from BC patients who underwent curative resection with informed consent between 2013 and 2014 at the Department of General Surgery, First Affiliated Hospital of Xi'an Jiaotong University. All tissues were collected immediately upon resection of the tumors in the operation theater, transported in liquid nitrogen, and then stored at −80°C. Another 336 breast cancer tissues which, were used for immunohistochemical analysis, were randomly collected from BC patients who underwent curative resection with informed consent between 2007 and 2010 at the Department of General Surgery, First Affiliated Hospital of Xi'an Jiaotong University. Tumor staging was based on the 6th edition of the tumor-node-metastasis (TNM) classification of the International Union Against Cancer. The clinicopathologic characteristics of the 336 breast cancer tissues are summarized in Supplemental Table 1. Study protocols were approved by the Hospital Ethics Committee of First Affiliated Hospital of Xi'an Jiaotong University, and written informed consent was obtained from patients based on the Declaration of Helsinki.

### Histological and immunohistochemical analysis

The normal human breast tissues, human tumor tissues, and tissues dissected from mice were fixed in 4% paraformaldehyde in phosphate-buffered saline (PBS) overnight and subsequently embedded in paraffin wax. Sections cut at a thickness of 4 μm were stained with hematoxylin and eosin for histological analysis. Immunohistochemical analysis was performed for different markers in these arrays as described previously [16]. The proportion of stained cells (lower, <30% staining; higher, ≥30% staining) was semiquantitatively determined following published protocols.

### Cell culture

BC cells (ATCC, Manassas, VA, USA) were cultured under the following conditions: MDA-MB-468, BT-20, Hs578T, UACC812 and MDA-MB-231 cell lines were cultured using 10% fetal bovine serum (Cat#10099–141, Invitrogen, Carlsbad, CA) in either RPMI-1640 (Cat#C11875, Invitrogen). T47D, AU565, and MDA-MB-435S cell lines were cultured using 10% fetal bovine serum (Invitrogen) in Dulbecco's modified Eagle medium (Cat#C11965, Invitrogen). Cell culture was according to manufacturer's protocol. All the cell lines were grown at 37°C in a 5% CO_2_/95% air atmosphere and were revived every 3 to 4 months.

### Establishment of overexpression and knockdown cell lines

Retroviral construct containing human pBabe-*Cullin3* cDNA, pcDNA3.1-*BRMS1* cDNA and pSuper.retro.puro with shRNA against human *Cullin3* and si*BRMS1* were prepared as described previously [17]. The generation of retrovirus supernatants and transfection of breast cancer cells were conducted as described previously. The expression of Cullin3 and BRMS1 was confirmed by qRT-PCR and Western blotting analysis.

### Cell proliferation assay

Cells were seeded in 96-well plates in triplicate at densities of 1 × 10^3^ per well. Cell proliferation was monitored at desired time points using 3-(4, 5-dimethylthiazol-2-yl)-2, 5-diphenyltetrazolium bromide (MTT). In brief, the MTT assay was performed by adding 20 μl MTT (5 mg/ml) for 4 h. Light absorbance of the solution was measured at 570 nm on a microplate reader.

### Wound healing assay

Cells were seeded in 6-cm culture plates, and the cell monolayers were wounded by scratching with sterile plastic 200 μl micropipette tips and photographed using phase-contrast microscopy. The migration distance of each cell was measured after the photographs were converted to Photoshop files.

### Cell invasion and motility assay

Invasion of cells was measured in Matrigel (BD, Franklin Lakes, NJ, USA) -coated Transwell inserts (6.5 mm, Costar, Manassas, VA, USA) containing polycarbonate filters with 8-μm pores as detailed previously [18]. The inserts were coated with 50 μl of 1 mg/ml Matrigel matrix according to the manufacturer's recommendations. 2 × 10^5^ cells in 200 μl of serum-free medium were plated in the upper chamber, whereas 600 μl of medium with 10% fatal bovine serum were added to lower well. After 24 hrs incubation, cells that migrated to the lower surface of the membrane were fixed and stained. For each membrane, five random fields were counted at × 10 magnification. Motility assays were similar to Matrigel invasion assay except that the Transwell insert was not coated with Matrigel.

### Confocal immunofluorescence microscopy

Cell lines were plated on culture slides (Costar, Manassas, VA, USA). After 24 hrs, the cells were rinsed with PBS and fixed with 4% paraformaldehyde, and cell membrane was permeabilized using 0.5% Triton X-100. These cells were then blocked for 30 min in 10% BSA and then incubated with primary antibodies overnight at 4°C. After three washes in PBS, the slides were incubated for 1 hour in the dark with FITC-conjugated secondary antibodies (Invitrogen, Grand Island, NY, USA). After three further washes, the slides were stained with DAPI for 5 min to visualize the nuclei, and examined using a Carl Zeiss confocal imaging system (LSM 780) (Carl Zeiss, Jena, Germany).

### Western blotting

Cells were lysed in lysis buffer and total protein contents were determined by the Bradford method. 30 μg of lysis were separated by reducing SDS-PAGE and probed with specific antibodies. Blots were washed and probed with respective secondary peroxidase-conjugated antibodies, and the bands visualized by chemoluminescence (Amersham Biosciences).

### qRT-PCR

Total RNA was extracted using Trizol reagent and cDNA was synthesized using SuperScript II Reverse Transcriptase (Invitrogen). qRT-PCR and data collection were performed with an ABI PRISM 7900HT sequence detection system. The primers used for the amplification of the indicated genes are available upon request.

### Gene expression profiling

Total RNA quality and quantity were determined using Agilent 2100 Bioanalyzer and NanoDrop ND-1000. Affymetrix HU U133 plus 2.0 arrays were used according to manufacturer's protocol. The data were initially normalized by robust multiarray average (RMA) normalization algorithms in expression console software (Affymetrix). Significantly altered genes between Cullin3 overexpression and its control cells were considered by scatter plots and the genes up- and down-regulated ≥5-fold. Clustering analysis was done using gene list by Gene Cluster v3.0 software, and heat maps were visualized using Java TreeView v1.1.4r3 software. Gene set enrichment analysis was carried out using ConceptGen (http://conceptgen.ncibi.org/core/conceptGen/index.jsp). Gene sets were either obtained from the ConceptGen or from published gene signatures.

### *In vivo* tumor growth and metastasis

Nude mice were purchased from the Shanghai Slac Laboratory Animal Co. Ltd and maintained in microisolator cages. All animals were used in accordance with institutional guidelines and the current experiments were approved by the Use Committee for Animal Care. For subcutaneous inoculation, different numbers of tumor cells were resuspended in PBS medium with 50% Matrigel and inoculated subcutaneously into the 8-week-old nude mice. The tumors were measured weekly and the tumor volume was calculated according to the formula length × width^2^/2. The mice were killed six weeks after the inoculation. For metastasis assays, cells were resuspended in PBS at a concentration of 1 × 10^7^ cells ml^−1^. Cell suspension (0.1 ml) was injected into tail veins of nude mice. All of the mice were killed by CO_2_ 60 days after inoculation.

### Statistical analysis

Results were analyzed with SPSS13.0 statistical software. Correlation between Cullin3 expression and clinicopathologic parameters was evaluated using the Chi-square (χ^2^) test, and quantitative variables were analyzed by the independent *t* test. The survival probability was estimated by Kaplan-Meier method, and the comparison of survival curves between groups was done with the log-rank test. The statistical significance of the differences between mean values was determined by *P* < 0.05.

## SUPPLEMENTARY FIGURES AND TABLE


