# Supplementary material for: Cullin3 promotes breast cancer cells metastasis and epithelial-mesenchymal transition by targeting BRMS1 for degradation
Source: Oncotarget. 2015 Oct 16;6(39):41959–75. doi: 10.18632/oncotarget.5999 (PMC4747201; doi:10.18632/oncotarget.5999)
Supplement: Supplementary file 1 [file oncotarget-06-41959-s001.pdf]

SUPPLEMENTARY FIGURES AND TABLE

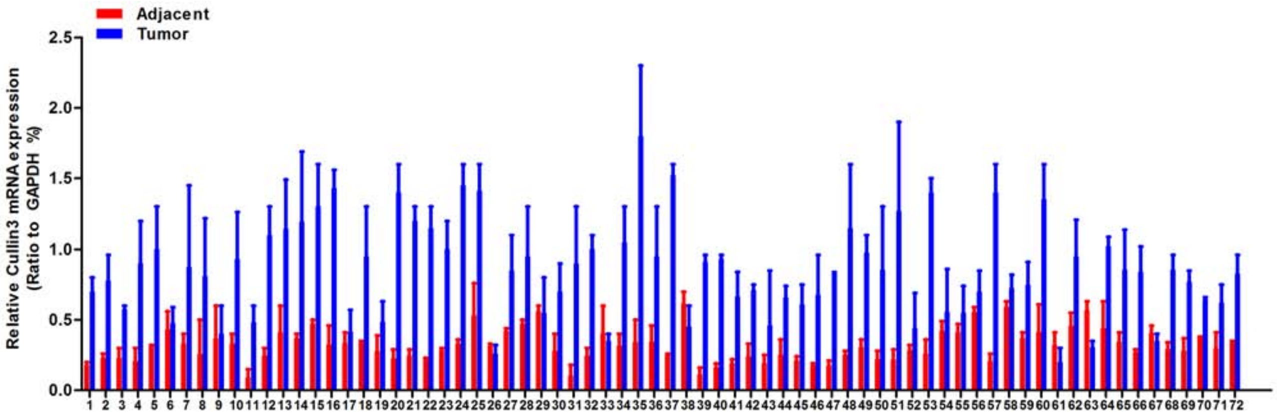

Supplementary Figure S1: The expression of Cullin3 mRNA was analyzed by qRT-PCR in 72 breast tumors and adjacent tissues.

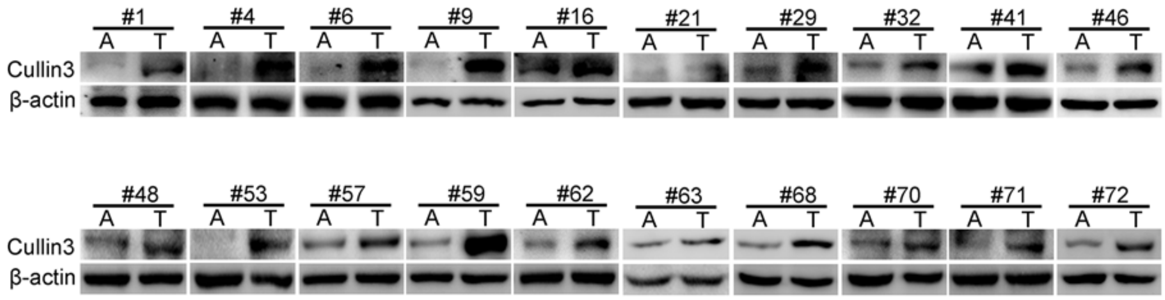

Supplementary Figure S2: The expression of Cullin3 protein was analyzed by Western blotting in 72 breast tumors and adjacent tissues.

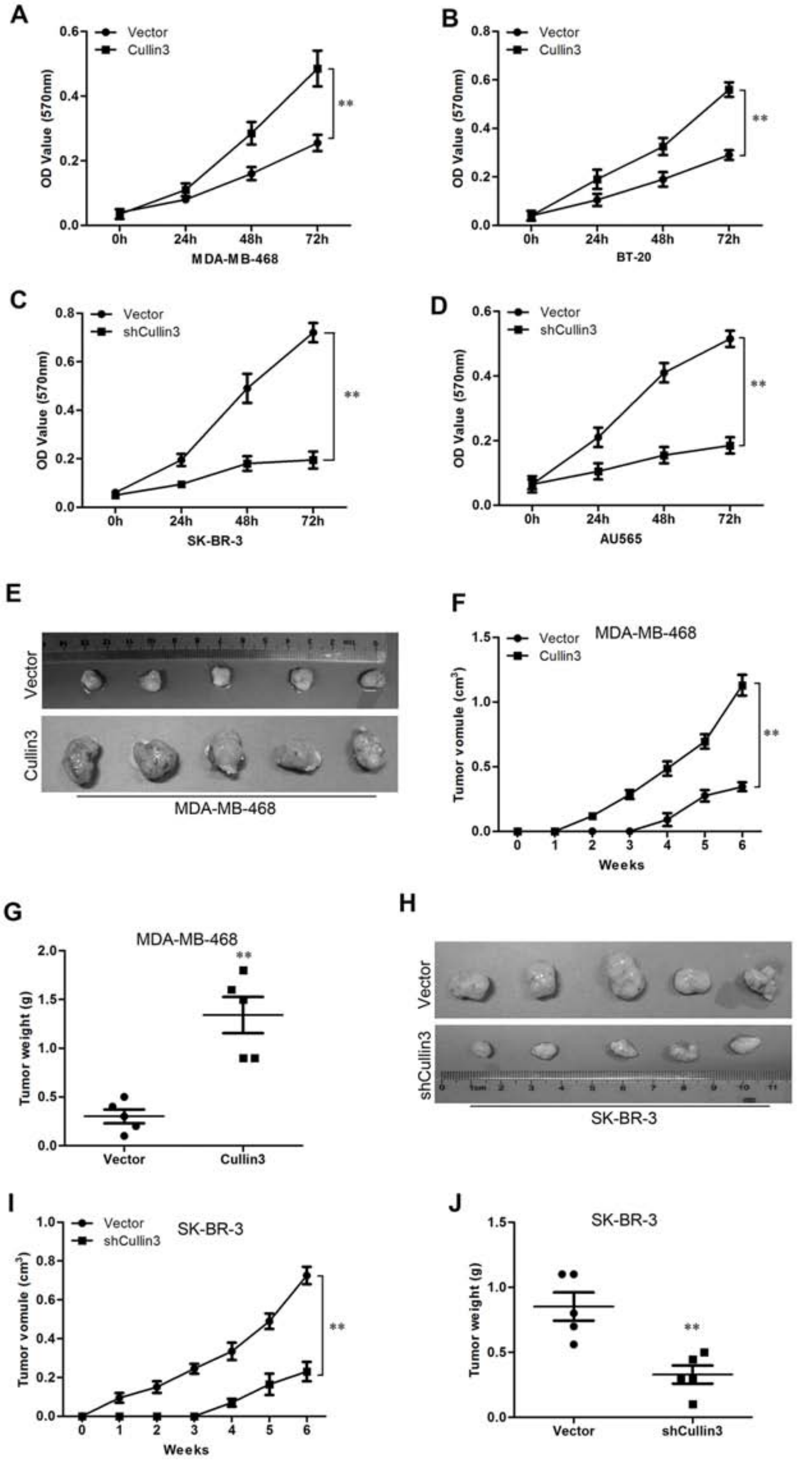

Supplementary Figure S3: Cullin3 promotes proliferative capacity of BC cells. **A.** MDA-MB-468-Cullin3 and its control vector cells proliferation was examined by MTT assays. **B.** BT20-Cullin3 and its control vector cells proliferation was examined by MTT assays. **C.** SK-BR-3-shCullin3 and its control vector cells proliferation was examined by MTT assays. **D.** AU565-shCullin3 and its control vector cells proliferation was examined by MTT assays. **E.** representative images of MDA-MB-468-Cullin3 and its control vector cells tumors by subcutaneous injection. **F.** growth curve of tumors formed by MDA-MB-468-Cullin3 and its control vector cells by subcutaneous injection. **G.** the weight of tumors formed by MDA-MB-468-Cullin3 and its control vector cells at harvest time. **H.** representative images of SK-BR-3-shCullin3 and its control vector cells tumors by subcutaneous injection. **I.** growth curve of tumors formed by SK-BR-3-shCullin3 and its control vector cells by subcutaneous injection. **J.** the weight of tumors formed by SK-BR-3-shCullin3 and its control vector cells at harvest time. $^{**}P < 0.01$ is based on the Student *t* test. All results are from three independent experiments. Error bars, SD.

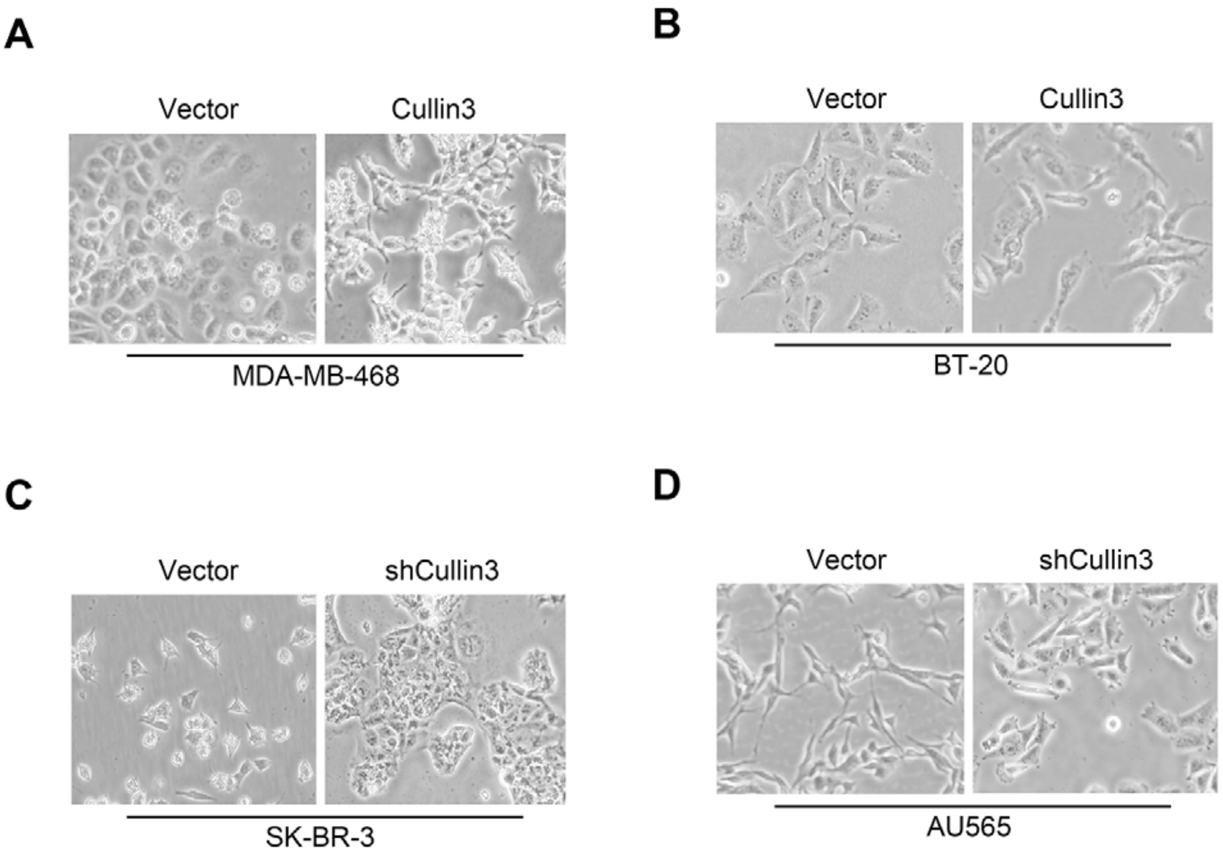

Supplementary Figure S4: Representative phase-contrast images of MDA-MB-468 **A.** and BT-20 **B.** cells showed Cullin3 overexpression-modulated morphologic changes. Representative phase-contrast images of SK-BR-3 **C.** and AU565 **D.** cells showed Cullin3 knockdown-modulated morphologic changes.

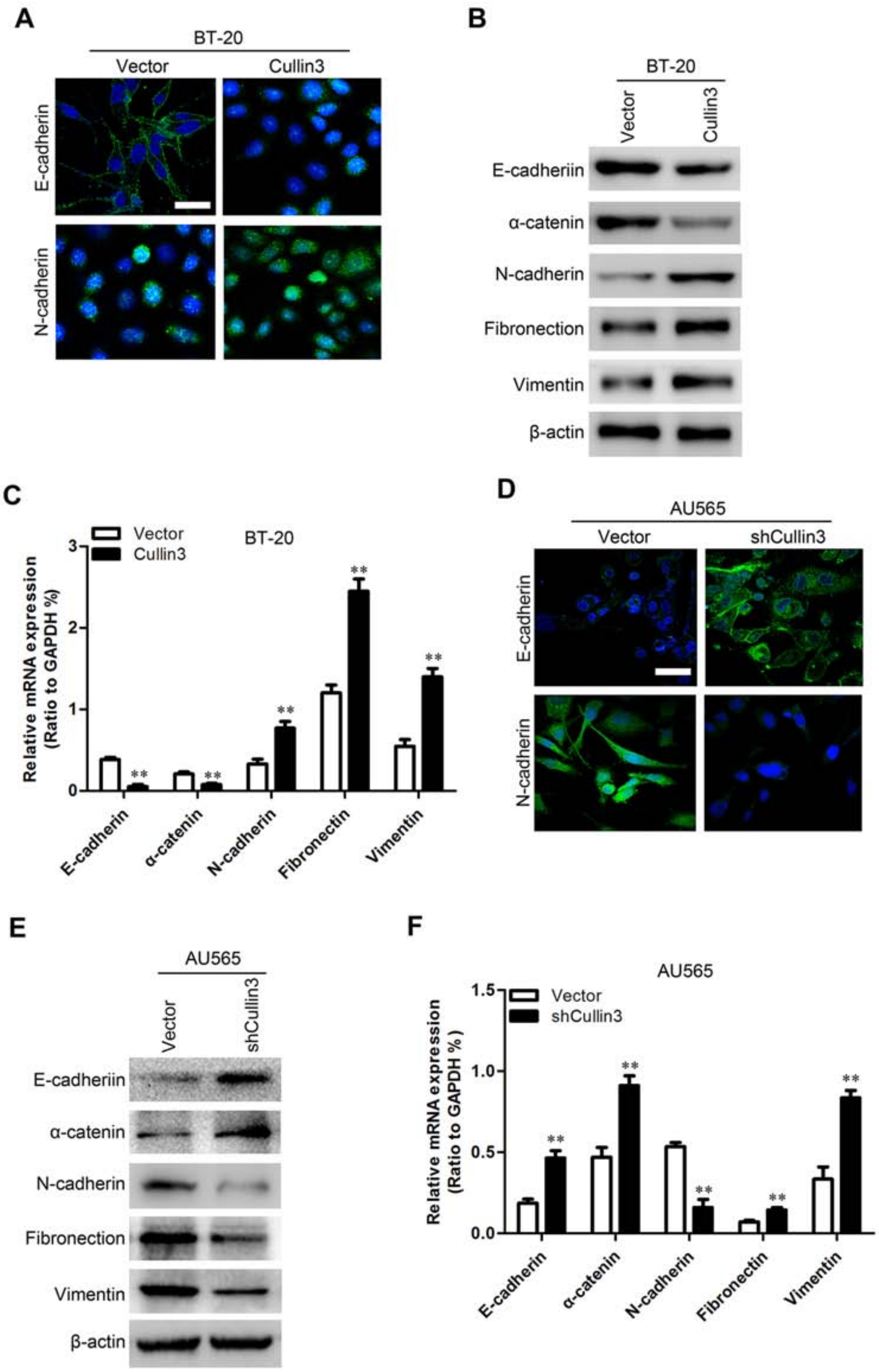

Supplementary Figure S5: Cullin3 regulates the transition between epithelial and mesenchymal phenotypes in BC cells. A. expression of E-cadherin and N-cadherin was analyzed by immunofluorescence stains in BT-20-Cullin3 and its control cells. **B.** expression of epithelial and mesenchymal marker was analyzed by Western blotting in BT-20-Cullin3 and its control cells. **C.** expression of epithelial and mesenchymal marker was analyzed by qRT-PCR in BT-20-Cullin3 and its control cells. **D.** expression of E-cadherin and N-cadherin was analyzed by immunofluorescence stains in AU565-shCullin3 and its control cells. **E.** expression of epithelial and mesenchymal marker was analyzed by Western blotting in AU565-shCullin3 and its control cells. **F.** expression of epithelial and mesenchymal marker was analyzed by qRT-PCR in AU565-shCullin3 and its control cells. $^{**}P < 0.01$ is based on the Student *t* test. All results are from three independent experiments. Error bars, SD.

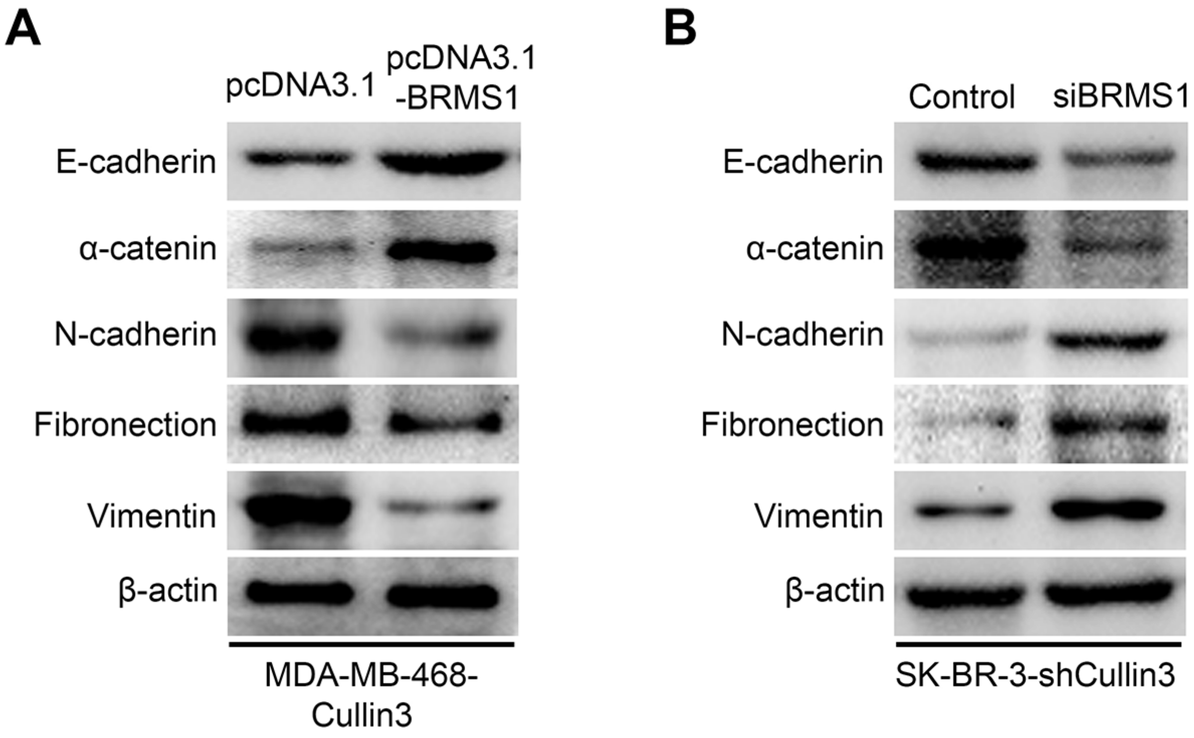

Supplementary Figure S6: BRMS1 is a mediator for Cullin3-induced NF-κB, Twist and FZD10 upregulation. A. the expression of NF-κB, Twist and FZD10 were analyzed by Western blotting in BRMS1 overexpressed MDA-MB-468-Cullin3 cells. **B.** the expression of NF-κB, Twist and FZD10 were analyzed by Western blotting in BRMS1 knocking down SK-BR-3-shCullin3 cells.

Supplementary Table S1: Association between Culllin3 expression and clinicopathological factors in 336 breast cancer patients

Characteristics	Group	Cullin3 negative, *n* (%)		Cullin3 positive, *n* (%)		*P* value
Age (y)	<35	7	(20.5)	53	(17.5)	.334
	≥ 35	27	(79.5)	249	(82.5)	
Tumor Size (cm)	≤ 2	11	(45.8)	228	(75.7)	.008*
	>2	23	(54.2)	74	(24.3)	
Lymph node metastasis	Negative	16	(45.1)	21	(7.6)	.002*
	Positive	18	(54.9)	281	(92.4)	
Histological grade	1,2	22	(64.7)	163	(53.9)	.564
	3	12	(35.3)	139	(46.1)	
Estrogen receptor	Negative	19	(55.9)	155	(51.3)	.687
	Positive	15	(44.1)	147	(48.7)	
Progesterone receptor	Negative	23	(67.6)	186	(61.2)	.087
	Positive	11	(32.4)	116	(38.8)	
Her-2	Negative	26	(76.5)	243	(80.1)	.661
	Positive	8	(23.5)	59	(19.9)	
Bcl-2	Negative	14	(41.2)	101	(33.4)	.714
	Positive	20	(58.8)	201	(66.6)	
Ki-67	<10%	23	(67.6)	235	(77.8)	.122
	≥ 10%	11	(32.4)	67	(22.2)	
P53	<50%	27	(79.4)	239	(79.1)	.614
	≥ 50%	7	(20.6)	63	(20.9)	
Chemotherapy	No	6	(17.6)	32	(10.6)	.301
	Yes	28	(82.4)	272	(89.4)	
Hormonal Therapy	No	2	(5.9)	45	(14.9)	.682
	Yes	32	(94.1)	257	(85.1)	
